# A Signature of 14 Long Non-Coding RNAs (lncRNAs) as a Step towards Precision Diagnosis for NSCLC

**DOI:** 10.3390/cancers14020439

**Published:** 2022-01-16

**Authors:** Anetta Sulewska, Jacek Niklinski, Radoslaw Charkiewicz, Piotr Karabowicz, Przemyslaw Biecek, Hubert Baniecki, Oksana Kowalczuk, Miroslaw Kozlowski, Patrycja Modzelewska, Piotr Majewski, Elzbieta Tryniszewska, Joanna Reszec, Zofia Dzieciol-Anikiej, Cezary Piwkowski, Robert Gryczka, Rodryg Ramlau

**Affiliations:** 1 Department of Clinical Molecular Biology, Medical University of Bialystok, 15-269 Bialystok, Poland; jacek.niklinski@umb.edu.pl (J.N.); radoslaw.charkiewicz@umb.edu.pl (R.C.); oksana.kowalczuk@umb.edu.pl (O.K.); 2 Center of Experimental Medicine, Medical University of Bialystok, 15-369 Bialystok, Poland; 3 Biobank, Medical University of Bialystok, 15-269 Bialystok, Poland; piotr.karabowicz@umb.edu.pl (P.K.); patrycja.modzelewska@umb.edu.pl (P.M.); joasia@umb.edu.pl (J.R.); zofia.dzieciol@umb.edu.pl (Z.D.-A.); 4 Faculty of Mathematics and Information Science, Warsaw University of Technology, 00-662 Warsaw, Poland; przemyslaw.biecek@pw.edu.pl (P.B.); hubert.baniecki.stud@pw.edu.pl (H.B.); 5 Department of Thoracic Surgery, Medical University of Bialystok, 15-269 Bialystok, Poland; miroslaw.kozlowski@umb.edu.pl; 6 Department of Microbiological Diagnostics and Infectious Immunology, Medical University of Bialystok, 15-269 Bialystok, Poland; piotr.majewski@umb.edu.pl (P.M.); trynisze@umb.edu.pl (E.T.); 7 Department of Medical Pathomorphology, Medical University of Bialystok, 15-269 Bialystok, Poland; 8 Department of Rehabilitation, Medical University of Bialystok, 15-089 Bialystok, Poland; 9 Department of Thoracic Surgery, Poznan University of Medical Sciences, 60-569 Poznan, Poland; cpiwkow@ump.edu.pl; 10 Department of Oncology, Poznan University of Medical Sciences, 60-569 Poznan, Poland; robert-gryczka@wp.pl (R.G.); rodrygramlau@ump.edu.pl (R.R.)

**Keywords:** lncRNA, lung cancer, diagnosis, biomarkers, epigenetics

## Abstract

**Simple Summary:**

Although the biological function of lncRNAs has not been fully elucidated, we know that the aberrant expression of lncRNAs can drive the cancer phenotype. Therefore, a growing area of research is focusing on lncRNAs as putative diagnostic biomarkers and therapeutic targets. The aim of the study was the appraisal of the diagnostic value of 14 differentially expressed lncRNA in the early stages of NSCLC. We established two classifiers. The first recognized cancerous from noncancerous tissues, the second successfully discriminated NSCLC subtypes (LUAD vs. LUSC). Our results indicate that the panel of 14 lncRNAs can be a promising tool to support a routine histopathological diagnosis of NSCLC.

**Abstract:**

LncRNAs have arisen as new players in the world of non-coding RNA. Disrupted expression of these molecules can be tightly linked to the onset, promotion and progression of cancer. The present study estimated the usefulness of 14 lncRNAs (HAGLR, ADAMTS9-AS2, LINC00261, MCM3AP-AS1, TP53TG1, C14orf132, LINC00968, LINC00312, TP73-AS1, LOC344887, LINC00673, SOX2-OT, AFAP1-AS1, LOC730101) for early detection of non-small-cell lung cancer (NSCLC). The total RNA was isolated from paired fresh-frozen cancerous and noncancerous lung tissue from 92 NSCLC patients diagnosed with either adenocarcinoma (LUAD) or lung squamous cell carcinoma (LUSC). The expression level of lncRNAs was evaluated by a quantitative real-time PCR (qPCR). Based on Ct and delta Ct values, logistic regression and gradient boosting decision tree classifiers were built. The latter is a novel, advanced machine learning algorithm with great potential in medical science. The established predictive models showed that a set of 14 lncRNAs accurately discriminates cancerous from noncancerous lung tissues (AUC value of 0.98 ± 0.01) and NSCLC subtypes (AUC value of 0.84 ± 0.09), although the expression of a few molecules was statistically insignificant (SOX2-OT, AFAP1-AS1 and LOC730101 for tumor vs. normal tissue; and TP53TG1, C14orf132, LINC00968 and LOC730101 for LUAD vs. LUSC). However for subtypes discrimination, the simplified logistic regression model based on the four variables (delta Ct AFAP1-AS1, Ct SOX2-OT, Ct LINC00261, and delta Ct LINC00673) had even stronger diagnostic potential than the original one (AUC value of 0.88 ± 0.07). Our results demonstrate that the 14 lncRNA signature can be an auxiliary tool to endorse and complement the histological diagnosis of non-small-cell lung cancer.

## 1. Introduction

Comprehensive analysis of the human genome unraveled far more transcriptionally active regions than had been previously anticipated. Contrary to expectations, the vast majority of the RNAs (over 80%) could neither be described as protein-coding nor considered “transcriptional noise”. This predominant bulk of the transcriptome has been named non-coding RNA (ncRNA) and with its regulatory capacities, has emerged as one of the frontline molecular players in a variety of biological phenomena. Non-coding RNAs are a heterogeneous population, including short ncRNA, middle-size ncRNA and long ncRNA. Over the last decade, researchers’ attention has focused mainly on short non-coding RNA (miRNA) but long non-coding RNA (lncRNA) has gradually gained importance [1].

According to LNCipedia, 56,946 lncRNA genes encoding 127,802 transcripts have been annotated [2]. The molecules are a part of the cellular machinery that regulates gene expression at the epigenetic, transcriptional, and posttranscriptional levels (e.g., histone modification, DNA methylation, chromatin looping, RNA maturation and transport, and protein synthesis). Mechanistically, lncRNAs can interact with transcription factors and RNA-binding proteins or even create molecular scaffolds to recruit different effectors [3,4]. Some lncRNAs have been classified into competing endogenous RNAs (ceRNAs). They form “miRNA sponges” to indirectly activate the miRNA target genes [5]. 

Differentially expressed lncRNAs (DElncRNAs) can be associated with cancer initiation, promotion, and progression [6,7]. Their expression depends on the stage of the disease and affects key pathophysiological pathways. Therefore, aberrantly expressed lncRNAs are potential biomarkers and a novel class of druggable targets. With reference to lung cancer, deep RNA-sequencing led to the finding of 6606, 856, and 170 aberrantly expressed lncRNAs to distinguish tumor from normal samples in non-small cell lung cancer (NSCLC), adenocarcinoma (LUAD), and lung squamous cell carcinoma (LUSC), respectively [8]. Further, functional studies of NSCLC cells revealed that, DElncRNAs deregulate multiple molecular pathways influencing invasiveness, migration, and metastasis [9]. For instance, lncRNA LINC01234 is involved in promotion of metastasis by two regulatory axes: miR-340-5p/miR-27b-3p in the cytoplasm; and EZH2, LSD1, and BTG2 in the nucleus [10]. Another lncRNA NNT-AS1, through NNT-AS1/miR-3666/E2F2, leads to lung cancer progression [11]. The proliferation of NSCLC cells and inhibition of apoptosis are connected with binding lncRNA SNHG20 to miR-197 as a part of downregulation of the Wnt/β-catenin signaling pathway [12]. 

Apart from that, studies have moved into mRNA-miRNA-lncRNA networks to search for prognostic and/or predictive biomarkers. Wang M et al. [13] created a triple network containing 41 lncRNAs, 192 miRNAs, and 775 mRNAs. They concluded that lncRNA EPB41L4A-AS1 is linked with the onset and prognosis of NSCLC and has the potential to become a therapeutic target. Wang X et al. [14] noticed that six mRNA (EGLN3, CCNB1, FOXG1, COL1A1, E2F7, and PFKP), three miRNA (miR-31, miR-144, and miR-192) and 16 lncRNA (AC080129.1, AC100791.1, AL163952.1, AP000525.1, AP003064.2, C2orf48, C10orf91, FGF12-AS2, HOTAIR, LINC00518, LNX1-AS1, MED4-AS1, MIG31HG, MUC2, TTTY16, and UCA1) are connected with overall survival of NSCLC patients. Huang Y et al. [15] described LINC00665-miR-let-7b-CCNA2 as being connected with the prognosis of LUAD.

It is also believed that the development of the robust diagnostic DElncRNA signature could support the routine histopathological examination of patients’ samples. Lin Y et al. [16] showed that two circulating lncRNAs: SNHG1 and RMRP distinguished lung cancer patients from cancer-free controls. Similarly, Yuan S et al. [17] constructed a panel of four circulating lncRNA (RMRP, NEAT1, TUG1, and MALAT1) that improved the diagnostic capacity in both LUAD and LUSC.

Despite advances in the understanding of the cancer genome, a great number of lncRNAs remain “the dark matter”. The biological functions of lncRNAs and underlying molecular mechanisms have not been fully revealed. Furthermore, there is a lack of strong evidence advocating that lncRNAs can be translated from bench to bedside [4]. To provide novel insight to precision diagnosis, we evaluated the expression of 14 lncRNAs (HAGLR, ADAMTS9-AS2, LINC00261, MCM3AP-AS1, TP53TG1, C14orf132, LINC00968, LINC00312, TP73-AS1, LOC344887, LINC00673, SOX2-OT, AFAP1-AS1, and LOC730101) in the early stages of non-small-cell lung cancer (NSCLC). Based on the PubMed database, we chose lncRNAs that (1) were shown to be differentially expressed in NSCLC; (2) were detected to be expressed in cell lines or tissues of NSCLC; (3) were noticed to have a potential role in the pathogenesis; and (4) were shown to be expressed by the established molecular methods (e.g., microarray, quantitative PCR). The expression level of selected molecules was measured by a quantitative polymerase chain reaction (qPCR). Then, the acquired Ct and delta Ct values were used to build a logistic regression classifier that successfully discriminated tumor from non-tumor tissue. However, in a more complex task of distinguishing NSCLC subtypes, a gradient boosting decision tree classifier (GBDT) was used to create the final simplified regression model based on four lncRNAs (AFAP1-AS1, SOX2-OT, LINC00261, and LINC00673) that was found to be a robust predictor of NSCLC subtype (AUC value of 0.88 ± 0.07).

## 2. Materials and Methods

### 2.1. Patients and Samples

The study was conducted, in the frame of the Polish STRATEGMED-2 and MINIATURA-2 projects, on 92 paired snap-frozen tumors and matched unaffected lung tissue collected from the lungs of IA-IIB NSCLC patients in the Department of Thoracic Surgery Medical University of Bialystok and in the Department of Thoracic Surgery Poznan University of Medical Sciences. None of the patients was treated with chemo- or radiotherapy before surgery. The informed consent for specimen collection and clinicopathological data processing was signed. The study design was approved by the Bioethics Committee of the Medical University of Bialystok (ethical approval codes: R-I-002/357/2014 and R-I-002/343/2018). Prior to RNA extraction, the cross-sections of frozen tissue samples were stained with hematoxylin-eosin and evaluated by a pathologist (J.R.) to confirm the content of cancer cells. Tumor specimens with a high percentage of the malignant cells (above 70% of tumor cells on a microscopic section) and normal lung epithelium without metaplasia or dysplasia were used for downstream application (tumor tissue and normal tissue, respectively). 

### 2.2. RNA Extraction and Quantitative Real-Time PCR

Total RNA, including the fraction of lncRNA, was isolated from tumor and adjacent normal lung tissue using the mirVana™ PARIS™ Kit (Thermo Fisher Scientific, Carlsbad, CA, USA), according to the manufacturer’s protocol. The method comprises an organic extraction followed by immobilization of RNA on glass-fiber filters. RNA concentration was measured on NanoDrop 2000c (Thermo Fisher Scientific, Carlsbad, CA, USA). RNA Integrity Number (RIN) was evaluated on a 2100 Bioanalyzer (Agilent, Santa Clara, CA, USA). The samples with RIN below six were automatically excluded. TR2 First Strand Kit (Qiagen, Germantown, MD, USA) was used for cDNA synthesis and for elimination of genomic DNA from RNA elutes, following producer’s recommendations. The level of expression of 14 lncRNAs and a reference gene (GAPDH) was accessed by quantitative Real-Time PCR (qPCR). The assays were designed against the combined NCBI RefSeq and Ensembl GENCODE database and guaranteed high qPCR efficiency. LncRNAs characteristics were summarized in Table 1. The amplification was performed in triplicates with LightCycler 480 (Roche, Basel, Switzerland) according to RT2 lncRNA qPCR Assays protocol (Qiagen, Germantown, MD, USA). To increase the accuracy and repeatability of pipetting, Agilent Bravo Automated Liquid Handling Platform (Agilent, Santa Clara, CA, USA) was applied. The variation between qPCR runs was compensated by the application of the interplate calibrator (IPC). Raw data (Ct), after IPC correction, was normalized according to the formula: dCt = Ct lncRNA gene—Ct reference gene (GAPDH). Relative quantification (RQ) was calculated as follows: 2 ^ (−ddCt) where: ddCt = dCt tumor—dCt normal.

### 2.3. Statistical Analysis

The differences in lncRNA expressions between the tumor and unaffected lung tissues were analyzed with the Wilcoxon matched-pairs signed-ranks test. The differences in lncRNA expression between NSCLC histological subtypes (LUAD tumor tissue vs. LUSC tumor tissue) were calculated with the Kruskal–Wallis rank tests. The Wilcoxon rank-sum (Mann–Whitney U) or Kruskal–Wallis rank tests were used to analyze the associations between clinicopathological variables and lncRNAs expression. After the Bonferroni correction, *p* ≤ 0.003 was considered to indicate statistically significant differences. The statistical analyses were performed using STATA/SE 11.1 software (Stata Corporation, College Station, TX, USA). 

Two binary logistic regression classifiers were established to appraise the diagnostic value of 14 lncRNAs. The first was created to differentiate cancerous and noncancerous samples. The latter has focused on the discrimination of LUSC tumor tissue from LUAD tumor tissue. In both models, as an input, Ct and delta Ct values were used. The models were evaluated with a fivefold stratified cross-validation. Samples were randomly assigned to the training sets and test sets. In each set, the classes were balanced. The random assignations to sets were repeated 100 times in total. For each step of cross-validation, the accuracy, recall, f1, a receiver operating characteristic (ROC) curve, and the area under the ROC (AUC) were established and results were expressed as mean and 95% CI. 

In order to validate the logistic regression metrics for discriminating between LUSC and LUAD, a more flexible gradient boosting decision tree classifier was built. The model was evaluated accordingly and then analyzed with Shapley Additive exPlanations (SHAP) [35,36] to quantify the importance of specific Ct and delta Ct values on the model’s predictions. Finally, the four most important variables were used as an input to establish a simplified logistic regression classifier, which was an improved version of the original model. The following Python packages were used in the statistical analysis: scikit-learn v1.0.1 [37] for the logistic regression classifier and model evaluation, lightgbm v3.3.1 [38] for the gradient boosting decision tree classifier, shap v0.40.0 [36], and dalex v1.4.1 [39] for the explanatory model analysis.

## 3. Results

### 3.1. Patient Characteristics

A total of 92 NSCLC patients, ages 44 to 81 years (M = 65; SD = 7,61) were included in the study. The majority of the patients were males (N = 62; 67%). Among the patients, 38 (41.30%) were diagnosed with adenocarcinoma and 54 (58.70%) with lung squamous cell carcinoma. According to TNM, all patients had early stages of cancer: IA—18 (19.6%) patients, IB—31 (33.7%) patients, IIA—14 (15.2%) patients and IIB—29 (31.5%) patients. The vast majority of patients were smokers 87 (94.6%) with mean value of pack years of 39.2 (SD = 21.36) and five (5.4%) patients identified as non-smokers (three females and two males) (Table 2).

### 3.2. Differences in lncRNAs Expression Levels between Tumor and Normal Tissue of the Lung

**Figure 1:**
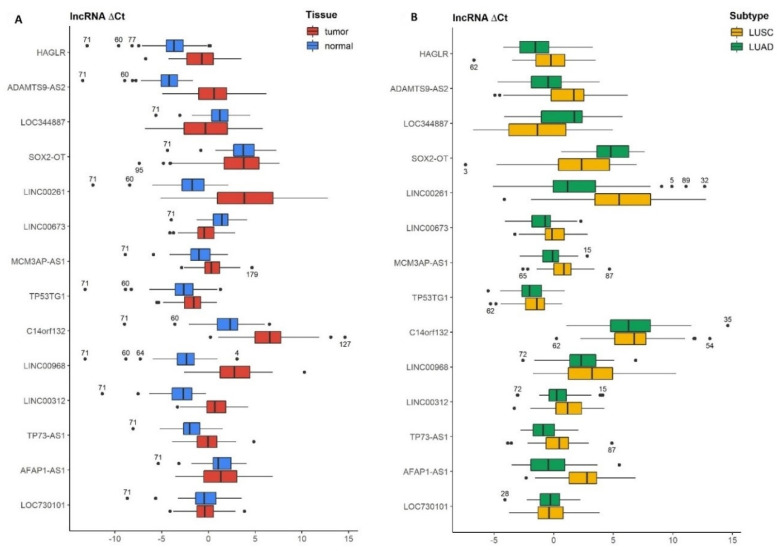
Visualization of differences in the delta Ct distributions across all lncRNAs. Additional numbers indicate the IDs of the furthest outlier patients in data: (**A**) Differences between the cancerous and noncancerous lung tissue. Two patients with extreme delta Ct values of multiple lncRNAs are observed (60 and 71); (**B**) Differences between the LUSC and LUAD subtypes of the tumor tissue. No patients with multiple extreme delta Ct values are observed.

Taking into account all cases of NSCLC, we found that 9 out of 14 lncRNAs (HAGLR, ADAMTS9-AS2, LINC00261, MCM3AP-AS1, TP53TG1, C14orf132, LINC00968, LINC00312, TP73-AS1) were downregulated in the tumor tissue compared to normal lung tissue; two lncRNAs (LOC344887and LINC00673) were upregulated and three lncRNAs (SOX2-OT, AFAP1-AS1, LOC730101) showed statistically insignificant differences (Table 3, Figure 1A). When analyzing LUSC tumor tissue vs. normal lung tissue, we observed a significant decrease in expression of 10 out of 14 lncRNAs (HAGLR, ADAMTS9-AS2, LINC00261, MCM3AP-AS1, TP53TG1, C14orf132, LINC00968, LINC00312, TP73-AS1, AFAP1-AS1); 3 lncRNAs (LOC344887, SOX2-OT, LINC00673) showed higher expression and lncRNA LOC730101 was statistically insignificant. In patients with LUAD, we reported that 7 lncRNAs out of 14 (HAGLR, ADAMANTS9-AS2, LINC00261, C14orf132, LINC00968, LINC00312, TP73-AS1) were downregulated in tumor in comparison to normal lung tissue; two lncRNAs were upregulated (LINC00673 AFAP1-AS1) and five lncRNAs (LOC344887, SOX2-OT, MCM3AP-AS1, TP53TG1, LOC730101) were statistically insignificant (Table 3).

### 3.3. Differences in lncRNAs Expression Levels between Lung Cancer Subtypes (LUAD Tumor vs. LUSC Tumor)

The expression of 10 out of 14 lncRNAs showed a significant association with the histopathological subtype of NSCLC. In LUAD tumor, compared to LUSC tumor, we found the increased expression of 8 lncRNAs (HAGLR, ADAMTS9-AS2, LINC00261, LINC00673, MCM3AP-AS1, LINC00312, TP73-AS1, AFAP1-AS1) and decreased expression of 2 lncRNA (LOC344887 and SOX2-OT) (Table 4, Figure 1B).

### 3.4. Correlation of lncRNAs Expression and Clinicopathological Characteristics of NSCLC Patients

The expression of 13 out of 14 lncRNAs was linked with the histological subtype of NSCLC. The majority of the studied lncRNAs have not shown any significant connection with the other clinicopathological features of patients. We observed that the expression of 3 out of 14 lncRNAs (LINC00673, MCM3AP-AS1, C14orf132) was associated with sex, the expression of 3 out of 14 lncRNAs (LOC344887, MCM3AP-AS1, LOC730101) was connected with death, and the expression of SOX2-OT was linked with the smoking status. Recurrence-free survival (RFS) and TNM have not been affected by the expression of any of 14 lncRNAs (Table 5).

### 3.5. Diagnostic Value of lncRNAs in NSCLC Patients Based on Logistic Regression Classifiers and Gradient Boosting Decision Tree

**Figure 2:**
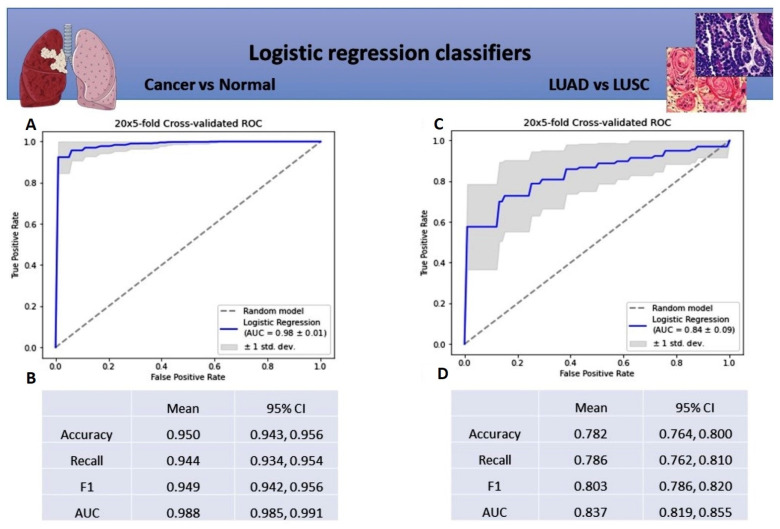
Metrics for logistic regression classifier for differentiating normal tissue from tumor tissue and LUSC tissue from LUAD tissue: (**A**) Mean ROC ± SD curve, and AUC for logistic regression classifier for differentiating normal tissue from cancer tissue; (**B**) Mean and 95% CI for precision, recall, f1-score, and AUC metrics for logistic regression classifier for differentiating normal tissue from cancer tissue; (**C**) Mean ROC ± SD curve, and mean AUC for logistic regression classifier for differentiating LUSC tissue from LUAD tissue; (**D**) Mean and 95% CI of precision, recall, f1-score metrics, and AUC for logistic regression classifier for differentiating LUSC tissue from LUAD tissue.

The first logistic regression model for cancerous and noncancerous tissue separation has shown a strong diagnostic potential (AUC value of 0.98 ± 0.01) (Figure 2A). All of the mean model metrics were above 0.9, indicating that lncRNAs successfully distinguished NSCLC tumor tissue from noncancerous lung tissue (Figure 2B). In the second logistic regression classifier for subtype discrimination, the mean area under the ROC curve (AUC) was 0.84 ± 0.09 (Figure 2C). All mean metrics were weaker than in the first model, but still above 0.75, suggesting that 14 lncRNAs taken together can potentially distinguish LUSC from LUAD tissue (Figure 2D). 

**Figure 3:**
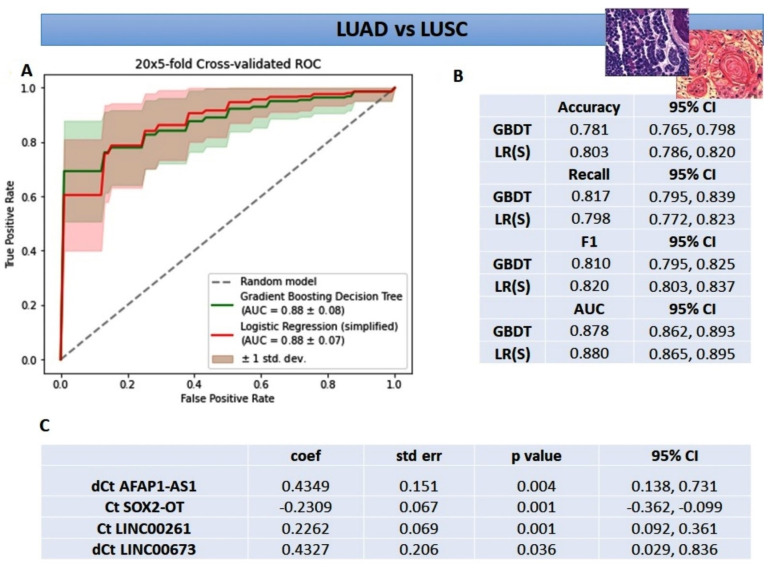
Metrics for gradient boosting decision tree and simplified logistic regression classifiers for differentiating LUSC tissue from LUAD tissue: (**A**) mean ROC ± SD curve, and mean AUC for the two classifiers; (**B**) mean and 95% CI of precision, recall, f1-score metrics, and AUC; (**C**) summary table with coefficients and significance for the simplified logistic regression classifier; GBDT—gradient boosting decision tree, LR(S)—logistic regression (simplified).

**Figure 4:**
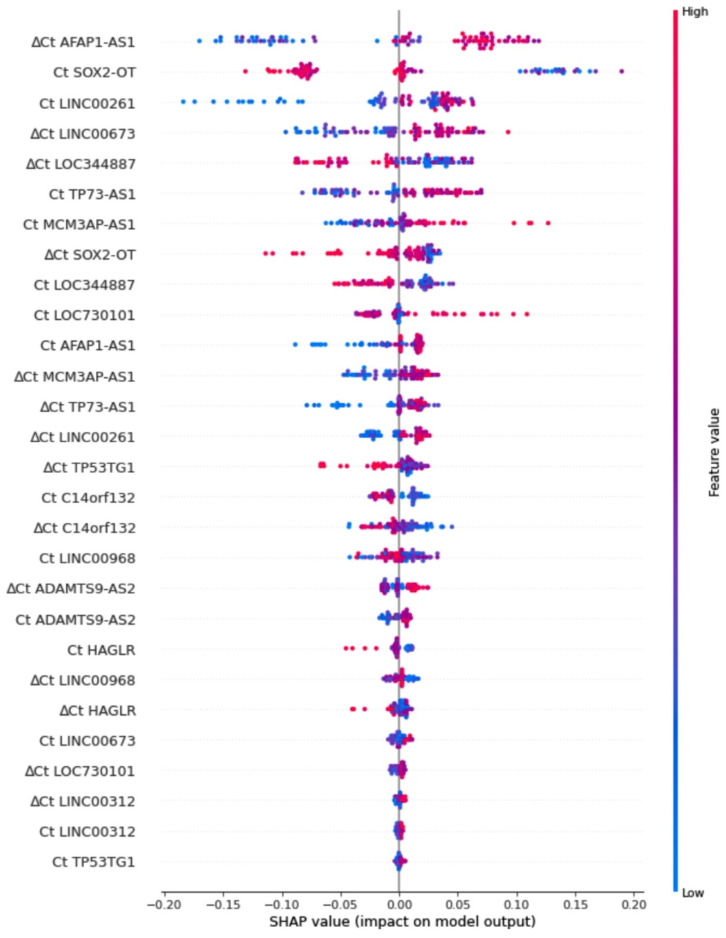
Shapley Additive Explanation for the gradient boosting decision tree classifier. The y-axis indicates a ranking of variables, Ct and delta Ct values of lncRNAs, sorted from the most important in the model (top) to the least important (bottom). The x-axis indicates an impact of a given variable on the model’s predictions; the SHAP values are sorted from the negative impact leading towards the LUAD subtype (class 0 on the left) to the positive impact leading towards the LUSC subtype (class 1 on the right). There are 92 points per row, one point per patient, where each indicates an attribution of a given variable to the probability model output. The color-axis indicates the variables’ values from low with blue to high with red. The visible distinction in colors between negative and positive SHAP values might be viewed as indicating a significant expression profile (up or down).

Improving the second logistic regression model a gradient boosting decision tree (GBDT) was created. This increased the AUC value up to 0.88 ± 0.08 with other mean metrics above 0.75 (Figure 3A,B). The better results were attributed to capturing the interactions between the variables and dealing better with the higher volume of noise in data (Figure 1B). Explanatory analysis of GBDT with SHAP highlighted the significance of variables for discriminating between LUSC and LUAD (Figure 4). From the explanation, it can be observed that the three most important variables used in the discrimination were delta Ct AFAP1-AS1, Ct SOX2-OT, and Ct LINC00261, while, for example, the expression of LINC00312, HAGLR, and ADAMTS9-AS2 were of little use to the model. Moreover, the expression profile of the most important lncRNAs, indicated with the red-to-blue and blue-to-red distinction, has been consistent with the results presented in Table 4 and Figure 1. The final simplified logistic regression model was built using only the top-four variables (Figure 3C) and showed the best diagnostic potential with the AUC value of 0.88 ± 0.07 and other mean metrics above 0.8 (Figure 3B).

Our findings pinpointed that the panel of 14 lncRNAs can be a novel and useful tool for both discrimination malignant from non-malignant lung tissue and for NSCLC subtyping.

## 4. Discussion

The focus of the study was on the possibility of the application of lncRNAs for precise diagnostic decision-making in NSCLC patients. We anticipated that the panel of 14 lncRNAs could distinguish between cancerous and noncancerous lung tissues as well as NSCLC subtypes (LUAD vs. LUSC). Therefore, we hypothesized that our lncRNA signature could be implemented along with histopathology to increase the accuracy of NSCLC diagnosis.

We identified a set of 11 lncRNAs (HAGLR, ADAMTS9-AS2, LINC00261, MCM3AP-AS1, TP53TG1, C14orf132, LINC00968, LINC00312, TP73-AS1, LOC344887, and LINC00673) dysregulated in NSCLC tumors compared with normal adjacent lung tissues, as well as a panel of 10 lncRNAs (HAGLR, ADAMTS9-AS2, LINC00261, LINC00673, MCM3AP-AS1, LINC00312, TP73-AS1, AFAP1-AS1, LOC344887, and SOX2-OT) differentially expressed between LUAD tumor and LUSC tumor. Our results were partially in line with those previously published.

We observed that five genes (HAGLR, ADAMTS9-AS2, TP73-AS1, LINC00261, and LINC00312) were downregulated in tumor vs. normal lung tissue (NSCLC, LUAD, LUSC); however, the expression was lifted in LUAD tumors in comparison to LUSC tumors. Our findings partially coincide with data from the literature. Yu H et al. [1] observed the decreased expression of ADAMTS9-AS2, LINC00261, LINC00312, HAGLR, and TP73-AS1 in NSCLC tumor vs. normal lung tissue. Guo X et al. [40], studying LUAD cases, noticed that HAGLR was downregulated in tumor vs. normal tissue. On the contrary, Li L et al. [41] showed that HAGLR was upregulated in NSCLC tissue compared with normal lung tissue, and the expression was associated with clinicopathological features of patients (tumor size, recurrence, TNM, stage, lymph node metastasis, and poor OS). In addition, HAGLR promoted NSCLC cell proliferation, migration, and invasion via several signaling pathways. Specifically, the molecule upregulated matrix metallopeptidase 9 (MMP9) by binding with miR-133b, aberrant regulated miR-147a/pRB [42], and downregulated p21 [43]. 

Our results were in opposition to papers arguing the TP73-AS1 upregulation in NSCLC tumor [44] and LUAD tumor [45] in comparison to normal adjacent lung tissue. Zhu D et al. [44] highlighted that upregulation of TP73-AS1 was associated with tumor size, TNM stage, lymph node metastasis (TNM), and poor prognosis and overexpression of p-21. During NSCLC progression TP73-AS1 targeted miR-449a-EZH2 axis [32], miR-27b-3p/LAPTM4B axis [33] or upregulated mir-21 [44]. In LUAD, TP73-AS1 activated PI3K/AKT [46] or Wnt/β-catenin signaling pathways [45]. 

The under-expressed ADAMTS9-AS2, LINC00261, and LINC00312 in our studies were in concordance with the publications underlining their tumor-suppressive potential. The experimentally induced overexpression of these molecules inhibited proliferation, invasion, and migration of tumor cells. Liu C et al. [19] augmented expression of ADAMTS9-AS2 that narrowed the size of lung tumor, arrested lung cancer cells in G1/G0 phase and promoted apoptosis. Moreover, ADAMTS9-AS2 inhibited miR-223-3p and activated TGFBR3. Guo C et al. [22] induced LINC00261 expression that regulated miR-1269a/FOXO1 axis. By the same token, Wang Z et al. [23] showed that LINC00261 suppressed miR-105/FHL1 axis, Shi J et al. [24] demonstrated that the molecule sponged miR-522-3p leading to the overexpression of SFRP2 that inhibited Wnt signaling and Liao J et al. [47] showed that LINC00261 restrained the epithelial–mesenchymal transition (EMT) of NSCLC via downregulating Snail. For the next molecule, LINC00312, the data were less straightforward. Tan Q et al. [48] presented that downregulation of LINC00312 was connected with advanced stage NSCLC, whereas, Tian Z et al. [49] concluded that it was linked with the I stage of LUAD.

We showed that C14orf132 and LINC00968 were downregulated in tumors (NSCLC, LUAD and LUSC) in comparison to matched normal lung tissue and not statistically significant in LUAD tumor vs. LUSC tumor. Yu H et al. [1] identified C14orf132 and LINC00968 as two of the most downregulated lncRNAs in NSCLC compared with normal lung tissue. The biological function of C14orf132 has not yet been revealed. LINC00968, on the other hand, was shown to be a part of a two-signaling axis in LUAD: miR-9-5p/CPEB3 [28] or miR-21-5p/SMAD7 [29]. The molecule also networked three miRNAs (miR-9, miR-22 and miR-4536) and two hub genes (PLK1 and XPO1) [50]. In LUSC, LINC00968 affected the outcome of patients via regulating the MAPK signaling pathway [51].

In our experiment, MCM3AP-AS1 and TP53TG1 were downregulated in NSCLC and LUSC tumors in comparison to normal lung tissue. However, in LUAD tumor vs. LUSC tumor, MCM3AP-AS1 was upregulated, while TP53TG1 did not show any statistically significant differences. Li X et al. [25] and Shen D et al. [26] noticed the elevated expression of MCM3AP-AS1 in NSCLC that accelerated cancer progression. The molecule targeted either miR-340-5p/KPNA4 axis or miR-195-5p/E2F3 axis. However, Xiao H et al. [27] detected the depleted expression of TP53TG1 in NSCLC and cell lines. The experimentally induced upregulation of TP53TG1 modulated the miR-18a/PTEN axis and enhanced cisplatin sensitivity and apoptosis of cancer cells.

In our study, LINC00673 was upregulated in tumors of all considered groups (NSCLC, LUSC, and LUAD) in comparison to adjacent noncancerous lung tissue as well as upregulated in LUAD tumor vs. LUSC tumors. The results overlapped with those previously published. LINC00673, a key regulator of signaling pathways, was shown to promote invasion and migration of NSCLC cells [52,53,54]. Ma C et al. [52] described that LINC00673 participated in epigenetic silencing of HOXA5 via binding EZH2. Lu W et al. [53] observed that the molecule sponging miR-150-5p modulated the expression of ZEB1, a key regulator of EMT, indirectly. Guan H et al. [54] indicated that LINC00673 led to activation of WNT/β-catenin signaling and consequently promoted aggressiveness of LUAD.

In our findings, LOC344887 was upregulated in NSCLC tumor and LUSC tumor compared with normal lung tissue and in LUSC tumor vs. LUAD tumor. The differences in the expression level between LUAD and normal lung tissue were not significant. Similarly considering SOX2-OT, we have not noticed significant differences in both NSCLC and LUAD tumor vs. normal lung tissue. However, the upregulation of SOX2-OT was statistically significant in LUSC tumor vs. both normal lung tissue and LUAD tumor (*p* < 0.003). In the literature, both LOC344887 and SOX2-OT were shown to be upregulated in NSCLC tissues compared with normal lung tissues. This state was connected with adverse outcomes (lymph node metastasis, advanced stage, and poorer differentiation) [20,21]. Wu B et al. [20] observed the increased expression of LOC344887 in NSCLC compared with adjacent normal tissue, which was connected with shorter overall survival time of patients. Chen Z et al. [55] presented that SOX2-OT modulated miR-30d-5p/PDK1 axis, which promoted proliferation, migration, and invasion of NSCLC cells. Herrera-Solorio AM et al. [21] noticed that SOX2-OT affected the expression of EGFR-pathway members AKT/ERK, which was linked with a poor clinical prognosis. 

In our studies, the expression of AFAP1-AS1 in the tumor vs. normal lung tissue was not statistically significant in NSCLC, downregulated in LUSC, and upregulated in LUAD. As far as only tumor tissues were concerned, AFAP1-AS1 was upregulated in LUAD vs. LUSC. According to the literature, AFAP1-AS1 was upregulated in both NSCLC and LUAD compared with matched non-tumor tissues. The overexpression of the gene was an unfavorable factor for patients [56,57]. Zhong Y et al. [58] experimentally confirmed that AFAP1-AS1 promoted lung cancer metastasis. The molecule interacted with SNIP1, inhibiting the degradation of c-Myc protein that activated the expression of ZEB1, ZEB2, and SNAIL. The downstream targets of c-Myc enhanced EMT and consequently the metastatic potential of the cells. Other studies have shown that AFAP1-AS1 repressed epigenetically of p21 [59] and HBP1 [60] and upregulated IRF7 and the RIG-I-like receptor signaling pathways [61]. In addition, Huang N et al. [62] observed that AFAP1-AS1 promoted cellular chemotherapy resistance indirectly. The molecule sponged miR-139-5p, leading to the upregulation of RRM2 that activated EGFR/AKT. Not only was the gene involved in epigenetic regulation, but also its own expression was silenced by DNA methylation [63].

Within 14 evaluated lncRNAs, only LOC730101 expression was not statistically significant in all of the studied groups. Liu L et al. [34] reported that the overexpression of LOC730101 increased the proliferative phenotype of lung cancer cells and was positively correlated with CCND1 and CCNE1, the downstream targets of the Wnt/b-catenin signaling pathway.

The discrepancies between the literature and our study may arise from the tumor biology and different researchers’ approaches to the experiment layout. Lung cancer is highly heterogeneous, with the presence of inflammatory changes in the non-neoplastic surroundings [64]. Individual and population features also influence the tumor. To minimize the impact of variabilities on our studies, pathologically assessed fresh-frozen tissues were used. The samples enriched in malignant cells (ranged from 70 to 100%) were classified as “tumor” whereas those without tumor cells were classified as “normal.” In contrast to our experiment, the majority of studies utilized cell cultures, a homogenous population deprived of influences from the environment, which therefore does not directly mirror the dynamics of genetic changes observed inside the organs and tissues undergoing cancer genesis [19,42,44,45]. Another aspect that characterized our research was the exclusion of undifferentiated large cell carcinoma (LCLC) cases, due to low prevalence in the population (2.9–9%) and difficulties in obtaining a representative group [65]. There is no doubt that the pattern of gene expression in LCLC may be different from those in LUAD and LUSC and change the overall picture of gene expression in NSCLC. 

A further source of inconsistencies could be associated with the restriction of our experiment to the early stages of NSCLC (IA–IIB), whereas others extended their interest to more advanced stages (III–IV) [1,41]. In the course of tumorigenesis, the gene expression changes. LncRNAs with oncogenic potential are more than likely to be downregulated in the early stages and upregulated as the disease progresses and local or distant metastases occur. That is why our results might not be in line with those obtained by researchers considering different stages of NSCLC.

The two binary regression classifiers established by us, based on all 14 lncRNAs, distinguished cancerous from noncancerous lung tissues and NSCLC subtypes (AUC of 0.98 ± 0.01 and 0.84 ± 0.09, respectively) with high precision, even if the expression of a few molecules was statistically insignificant (SOX2-OT, AFAP1-AS1, and LOC730101 for tumor vs. normal tissue; and TP53TG1, C14orf132, LINC00968, and LOC730101 for LUAD vs. LUSC). To expand our knowledge on the importance of specific lncRNAs toward discriminating LUAD from LUSC tumor types, Shapley Additive Explanation of a gradient boosting decision tree was used. This one-step procedure of variable selection explaining a more complex classifier allowed us to build a very simple logistic regression model of high performance (AUC of 0.88 ± 0.07). The popularity of tree-based models, and especially interpreting their predictions, has seen a major impact across the medical domain [35]. The visible advantage of this approach presented in our study was accurately dealing with noisy data, which improved performance with an even more interpretable model, consisting of only 4 out of 28 variables (Ct and delta Ct values for all 14 lncRNAs). 

Since we were the first to construct classifiers using this particular set of long non-coding RNAs, it is impossible to refer to literature directly. Previously reported lncRNA signatures were mostly related to the prognoses and predictions of NSCLC. For instance, seven lncRNA signatures (APTR, DHRS4-AS1, ITGA9-AS1, LINC01137, LOC101927972, RPARP-AS1, and SH3BP5-AS1) [66], and six lncRNA signatures (LINC01287, SNAP25-AS1, LINC00470, AC104809.2, LINC00645, and LINC01010) [67] had prognostic value for lung cancer. The next six lncRNAs (LINC01819, ZNF649-AS1, HNF4A-AS1, FAM222A-AS1, LINC02323, and LINC00672) were independent predictors of tumor relapse in lung adenocarcinoma [68]. Two lncRNAs, TMPO-AS1 and C1orf132, affected the prognosis of LUAD patients [69], whereas, three lncRNAs (LINC02555, APCDD1L-DT and OTX2-AS1) were associated with LUSC patient survival [70].

In summary, our results demonstrate that 14 lncRNAs (HAGLR, ADAMTS9-AS2, LINC00261, MCM3AP-AS1, TP53TG1, C14orf132, LINC00968, LINC00312, TP73-AS1, LOC344887, LINC00673, SOX2-OT, AFAP1-AS1, and LOC730101) had the potential to separate cancerous from non-cancerous lung tissue as well as LUAD from LUSC in the early stages of NSCLC. We believe that the application of the panel of 14 lncRNAs can be a step towards a precise diagnosis for the early stages of NSCLC.

## 5. Conclusions

In the set of molecules we analyzed, 11 DElncRNAs successfully discriminated NSCLC tissue from corresponding adjacent non-cancerous tissue. The expression of 10 out of 14 lncRNAs was subtype-related (LUAD tumor or LUSC tumor). Using binary logistic regression classifiers and a tree-based model, we not only distinguished tumors from non-tumor tissues but also confirmed histological subtyping. Our results showed that the 14 lncRNA signatures can be a promising auxiliary tool to endorse and complement histopathological assessment. Nevertheless, a validation step in an independent group of patients is required. The expansion of future projects to advanced stages of NSCLC is also reasonable, given that the changes in lncRNA expression can be cancer stage-related.

## Figures and Tables

**Table 1:** The brief characteristics of 14 lncRNA selected for the experiment.

Symbol	Detected Transcripts	Qiagen ID	Name	Target	Mechanism of Tumorigenesis	Biological Activity	Ref.
HAGLR	NR_033979	LPH04484A	HOXD Antisense Growth-Associated Long Non-Coding RNA	MMP9; miR-147a	cell proliferation (+); cell migration (+); cell invasion (+)	ceRNA(miR-133b); sponge	[18]
ADAMTS9-AS2	ENST00000460833	LPH14614A	ADAMTS9 Antisense RNA 2	TGFBR3	cell proliferation (−); cell migration (−); apoptosis rate (+)	ceRNA(miR-223-3p); sponge	[19]
LOC344887	ENST00000306399	LPH01284A	NmrA Like Redox Sensor 2, Pseudogene		lymph node metastasis (+); advance stage (+); poorer differentiation (+)		[20]
SOX2-OT	ENST00000410534	LPH15037A	SOX2 Overlapping Transcript	AKT/ERK, SOX2/GLI-1	resistance to (TKI)-erlotinib (+) resistance to cisplatin-based therapy (+), clinical prognosis (−); lung malignant phenotype (+)	interact with protein	[21]
LINC00261	NR_001558	LPH22443A	Long Intergenic Non-Protein Coding RNA 261	FOXO1; SFRP2; FHL1	growth (−); metastasis (−)	ceRNA(miR-1269a); ceRNA(miR-522-3p); ceRNA(miR-105); sponge	[22,23,24]
LINC00673	NR_036488	LPH00090A	Long Intergenic Non-Protein Coding RNA 673	EZH2, miR-150-5p, HOXA5, NCALD, KDM1A	oncogenic role (+), cell proliferation (+); cell invasion (+); cell migration (+); epithelial to mesenchymal transition (+)	histone modification; interact with protein; sponge	[18]
MCM3AP-AS1	NR_002776	LPH03207A	MCM3AP Antisense RNA 1	KPNA4, E2F3	angiogenesis (+); proliferation (+), migration (+), invasion (+)	ceRNA(miR-340-5p); ceRNA(miR-195-5p); sponge	[25,26]
TP53TG1	NR_015381	LPH01569A	TP53 Target 1	PTEN	cisplatin sensitivity (+); apoptosis (+)	ceRNA(miR-18a); sponge	[27]
C14orf132	ENST00000556728	LPH41601A	Chromosome 14 Open Reading Frame 132		downregulated in NSCLC		[1]
LINC00968	ENST00000499425	LPH15879A	Long Intergenic Non-Protein Coding RNA 968	CPEB3; SMAD7	cell migration (−); colony formation (−); EMT (−), metastasis (−)	ceRNA(miR-9-5p); ceRNA(miR-21-5p); sponge	[28,29]
LINC00312	NR_024065	LPH13268A	Long Intergenic Non-Protein Coding RNA 312	YBX1; HOXA5	Migration (+); invasion (+); VM (+); cell proliferation (+); apoptosis (+)	interact with protein	[30,31]
TP73-AS1	NR_033708	LPH06577A	TP73 Antisense RNA 1	EZH2; LAPTM4B	cancer progression (+)	ceRNA(miR-449a); ceRNA(miR-27b-3p)	[32,33]
AFAP1-AS1	ENST00000608442	LPH27887A	AFAP1 Antisense RNA 1	CDKN1A	cell proliferation (+); prognosis (−)	epigenetic regulation	[18]
LOC730101	ENST00000462729	LPH03357A	Uncharacterized LOC730101	CCND1, CCNE1, β-catenin	progression of cell cycle (+); cell proliferation (+); growth (+)		[34]

**Table 2:** Characteristic of patients including age, overall survival (OS), recurrence-free survival (RFS), and pack-years. Non-small cell lung cancer (NSCLC), squamous cell carcinoma (LUSC), adenocarcinoma (LUAD).

	**N = NSCLC**	**Mean**	**SD**	**25%**	**75%**
age	92	65.04	7.61	61.00	70.00
rfs	92	16.18	8.73	9.00	23.50
os	92	17.55	8.24	11.00	24.00
pack-years	92	39.20	21.36	29.00	49.00
	**N = LUSC**	**Mean**	**SD**	**25%**	**75%**
age	54	65.33	7.28	61.00	69.00
rfs	54	16.37	8.12	10.25	22.75
os	54	18.20	7.28	12.00	24.00
pack-years	54	40.31	20.39	28.50	48.75
	**N = LUAD**	**Mean**	**SD**	**25%**	**75%**
age	38	64.63	8.06	60.25	70.00
rfs	38	15.92	9.54	9.00	24.00
os	38	16.63	9.38	9.25	24.00
pack-years	38	37.61	22.61	30.25	49.00

**Table 3:** Expression of 14 lncRNA in tumor tissue vs. normal tissue; (**A**) non-small cell lung cancer (NSCLC); (**B**) squamous cell lung carcinoma (LUSC); (**C**) lung adenocarcinoma (LUAD).

**A**		**lncRNA Delta Ct Tumor**				**lncRNA Delta Ct Normal**				**lncRNA Delta Delta Ct**					
	**N**	**Mean**	**SD**	**25%**	**75%**	**Mean**	**SD**	**25%**	**75%**	***p* Value**	**Mean**	**SD**	**25%**	**75%**	**Expression Profile**
HAGLR	92	−0.73	2.04	−2.32	0.54	−3.73	2.06	−4.54	−2.68	<0.003	2.99	2.69	2.43	3.55	down
ADAMTS9-AS2	92	0.37	2.2	−1.08	2.02	−4.36	1.73	−5.07	−3.29	<0.003	4.73	2.85	4.14	5.32	down
LOC344887	92	−0.25	3.11	−2.61	2.06	1.11	1.59	0.34	2.12	<0.003	−1.37	3.22	−2.03	−0.7	up
SOX2-OT	92	3.14	3.1	1.65	5.43	3.72	1.77	2.68	4.92	0.1604	−0.58	3.17	−1.24	0.08	up
LINC00261	92	4.24	4.11	0.94	6.97	−1.86	2.17	−2.81	−0.46	<0.003	6.11	4.89	5.09	7.12	down
LINC00673	92	−0.33	1.49	−1.16	0.63	1.34	1.33	0.5	2.11	<0.003	−1.67	1.93	−2.07	−1.27	up
MCM3AP-AS1	92	0.4	1.36	−0.42	1.22	−1.04	1.7	−1.92	0.18	<0.003	1.44	2.26	0.97	1.91	down
TP53TG1	92	−1.75	1.31	−2.58	−0.83	−2.84	2.07	−3.57	−1.59	<0.003	1.09	2.23	0.63	1.56	down
C14orf132	92	6.66	2.66	4.96	7.77	2.01	2.19	0.92	3.18	<0.003	4.64	3.25	3.97	5.32	down
LINC00968	92	2.87	2.18	1.24	4.48	−2.54	2.1	−3.33	−1.5	<0.003	5.42	2.88	4.82	6.01	down
LINC00312	92	0.82	1.56	−0.18	1.88	−2.91	1.77	−3.92	−1.75	<0.003	3.73	2.35	3.24	4.21	down
TP73-AS1	92	−0.15	1.52	−1.21	0.92	−1.88	1.51	−2.67	−0.9	<0.003	1.73	1.96	1.33	2.14	down
AFAP1-AS1	92	1.33	2.37	−0.57	3.07	1.26	1.51	0.48	2.43	0.7688	0.07	2.81	−0.51	0.65	down
LOC730101	92	−0.38	1.48	−1.28	0.55	−0.37	1.79	−1.37	0.83	0.9488	−0.01	2.36	−0.49	0.48	up
**B**		**lncRNA Delta Ct Tumor**				**lncRNA Delta Ct Normal**				**lncRNA Delta Delta Ct**					
	**N**	**Mean**	**SD**	**0.25**	**0.75**	**Mean**	**SD**	**0.25**	**0.75**	***p* Value**	**Mean**	**SD**	**0.25**	**0.75**	**Expression Profile**
HAGLR	54	−0.33	1.99	−1.87	0.92	−4.18	2.27	−4.94	−2.93	<0.003	3.84	2.47	2.05	5.56	down
ADAMTS9-AS2	54	0.97	2.28	−0.48	2.53	−4.72	1.97	−5.39	−3.46	<0.003	5.7	2.9	4.03	7.27	down
LOC344887	54	−1.16	3.16	−3.74	1.12	1.11	1.57	0.41	2.2	<0.003	−2.26	3.24	−5.06	0.28	up
SOX2-OT	54	2	3.35	0.43	4.64	3.36	1.93	2.22	4.44	<0.003	−1.36	3.55	−3.17	0.97	up
LINC00261	54	5.49	3.82	3.41	8.02	−2.28	2.43	−3.58	−0.6	<0.003	7.78	4.6	4.34	10.15	down
LINC00673	54	0.02	1.37	−0.71	0.85	1.19	1.32	0.51	1.88	<0.003	−1.18	1.75	−2.4	0.07	up
MCM3AP-AS1	54	0.79	1.38	−0.01	1.44	−1.35	1.86	−2.01	−0.17	<0.003	2.14	2.29	0.63	3.54	down
TP53TG1	54	−1.65	1.3	−2.39	−0.79	−3.24	2.33	−4.13	−1.98	<0.003	1.59	2.37	−0.07	3.08	down
C14orf132	54	6.69	2.49	5.05	7.72	1.56	2.44	0.41	2.77	<0.003	5.13	3.05	3.11	6.63	down
LINC00968	54	3.21	2.34	1.26	4.92	−2.98	2.41	−3.44	−1.73	<0.003	6.19	3.04	4.04	8.12	down
LINC00312	54	1.09	1.55	0.08	2.33	−3.35	1.96	−4.26	−1.98	<0.003	4.44	2.48	2.78	5.73	down
TP73-AS1	54	0.26	1.61	−0.79	1.22	−2.17	1.6	−2.94	−1.12	<0.003	2.43	1.91	1.26	3.99	down
AFAP1-AS1	54	2.38	1.96	1.26	3.6	0.98	1.64	0.36	1.87	<0.003	1.39	2.35	−0.35	2.79	down
LOC730101	54	−0.34	1.64	−1.28	0.76	−0.7	1.94	−1.62	0.62	0.3175	0.35	2.55	−1.16	1.79	down
**C**		**lncRNA Delta Ct Tumor**				**lncRNA Delta Ct Normal**				**lncRNA Delta Delta Ct**					
	**N**	**Mean**	**SD**	**0.25**	**0.75**	**Mean**	**SD**	**0.25**	**0.75**	***p* Value**	**Mean**	**SD**	**0.25**	**0.75**	**Expression Profile**
HAGLR	38	−1.3	1.99	−2.66	−0.4	−3.08	1.51	−4.09	−2.21	<0.003	1.78	2.54	0.06	2.34	down
ADAMTS9-AS2	38	−0.49	1.77	−1.73	0.66	−3.85	1.17	−4.76	−3.05	<0.003	3.37	2.15	1.89	5.05	down
LOC344887	38	1.03	2.58	−1.09	2.39	1.12	1.63	0.33	1.7	0.7548	−0.09	2.77	−2.17	1.76	up
SOX2-OT	38	4.75	1.76	3.6	6.31	4.22	1.38	3.35	5.27	0.2541	0.52	2.12	−0.98	1.67	down
LINC00261	38	2.47	3.88	0.07	3.51	−1.26	1.57	−2.42	−0.39	<0.003	3.73	4.33	0.7	5.52	down
LINC00673	38	−0.82	1.52	−1.86	−0.22	1.54	1.33	0.54	2.41	<0.003	−2.36	1.98	−3.55	−0.86	up
MCM3AP-AS1	38	−0.15	1.16	−0.84	0.41	−0.6	1.36	−1.83	0.48	0.1378	0.45	1.81	−0.65	1.64	down
TP53TG1	38	−1.9	1.32	−2.7	−1.02	−2.29	1.47	−3.49	−1.38	0.1249	0.39	1.82	−0.7	1.62	down
C14orf132	38	6.61	2.92	4.93	8.33	2.65	1.58	1.87	3.39	<0.003	3.96	3.43	1.38	7.06	down
LINC00968	38	2.39	1.87	1.29	3.58	−1.92	1.35	−2.93	−0.84	<0.003	4.31	2.26	2.9	5.63	down
LINC00312	38	0.44	1.52	−0.32	1.1	−2.27	1.23	−3.14	−1.29	<0.003	2.71	1.7	1.49	3.72	down
TP73-AS1	38	−0.74	1.15	−1.69	0.08	−1.48	1.27	−2.38	−0.67	<0.003	0.75	1.58	−0.08	1.91	down
AFAP1-AS1	38	−0.16	2.09	−1.85	0.96	1.64	1.24	0.87	2.57	<0.003	−1.8	1.58	−3.38	0.18	up
LOC730101	38	−0.42	1.25	−1.18	0.5	0.09	1.44	−0.96	1.13	0.0951	−0.51	1.98	−0.42	1.35	up

**Table 4:** Expression of 14 lncRNA in LUAD tumor vs. LUSC tumor.

		lncRNA Delta Ct LUSC Tumor				lncRNA Delta Ct LUAD Tumor					lncRNA Delta Delta Ct					
	N	Mean	SD	0.25	0.75	N	Mean	SD	0.25	0.75	*p* Value	Mean	0.25	0.75	Expression Profile LUSC	Expression Profile LUAD
HAGLR	54	−0.34	2.00	−0.88	0.21	38	−1.30	2.00	−1.96	−0.65	<0.003	0.97	0.13	1.81	down	up
ADAMTS9-AS2	54	0.97	2.28	0.35	1.60	38	−0.49	1.77	−1.07	0.97	<0.003	1.46	0.58	2.34	down	up
LOC344887	54	−1.16	3.16	−2.02	−0.30	38	1.03	2.58	0.18	1.87	<0.003	−2.18	−3.41	0.95	up	down
SOX2-OT	54	2.00	3.35	1.09	2.92	38	4.75	1.76	4.17	5.33	<0.003	−2.74	−3.92	−1.56	up	down
LINC00261	54	5.49	3.82	4.45	6.54	38	2.47	3.88	1.19	3.74	<0.003	3.03	1.41	4.64	down	up
LINC00673	54	0.02	1.38	−0.36	0.39	38	−0.82	1.52	−1.32	−0.32	<0.003	0.84	0.23	1.44	down	up
MCM3AP-AS1	54	0.78	1.38	0.41	1.16	38	−0.15	1.16	−0.53	0.23	<0.003	0.93	0.39	1.48	down	up
TP53TG1	54	−1.65	1.30	−2.00	−1.29	38	1.90	1.32	−2.33	−1.46	0.2105	0.25	−0.30	0.80	down	up
C14orf132	54	6.68	2.49	6.00	7.36	38	6.61	2.92	5.66	7.57	0.5145	0.07	−1.05	1.20	down	up
LINC00968	54	3.21	2.34	2.57	3.85	38	2.39	1.87	1.78	3.01	0.0916	0.82	−0.91	1.72	down	up
LINC00312	54	1.09	1.55	0.67	1.52	38	0.43	1.52	−0.06	0.94	<0.003	0.66	0.01	1.31	down	up
TP73-AS1	54	0.26	1.61	−0.18	0.70	38	−0.74	1.15	−1.12	−0.37	<0.003	1.00	0.40	1.61	down	up
AFAP1-AS1	54	2.38	1.96	1.84	2.91	38	−0.16	2.09	−0.85	0.53	<0.003	2.54	1.69	3.38	down	up
LOC730101	54	−0.35	1.64	−0.79	0.10	38	−0.42	1.25	−0.83	−0.01	0.8249	0.08	−0.55	0.70	down	up

**Table 5:** Expression of lncRNAs and clinicopathological parameters of NSCLC patients.

*p* Value							
Symbol	N	Sex	Histology	Recurrence	Death	Smoking	TNM
HAGLR	92	0.2	<0.003	0.76	0.2	0.34	0.07
ADAMTS9-AS2	92	0.14	<0.003	0.77	0.21	0.19	0.26
LOC344887	92	0.72	<0.003	0.13	<0.003	0.06	0.18
SOX2-OT	92	0.34	<0.003	0.33	0.51	<0.003	0.22
LINC00261	92	0.19	<0.003	0.79	0.09	0.09	0.38
LINC00673	92	<0.003	<0.003	0.36	0.09	0.7	0.25
MCM3AP-AS1	92	<0.003	<0.003	0.15	<0.003	0.49	0.15
TP53TG1	92	0.15	<0.003	0.23	0.06	0.9	0.08
C14orf132	92	<0.003	0.07	0.56	0.17	0.9	0.17
LINC00968	92	0.08	<0.003	0.29	0.06	0.47	1
LINC00312	92	0.12	<0.003	0.1	0.1	0.78	0.91
TP73-AS1	92	0.07	<0.003	0.2	0.05	0.78	0.1
AFAP1-AS1	92	0.15	<0.003	0.4	0.26	0.09	0.32
LOC730101	92	0.19	<0.003	0.06	<0.003	0.11	0.2

## Data Availability

The datasets analyzed during the current study are available from the corresponding author on reasonable request.
